# Resveratrol Protects Rats from Aβ-induced Neurotoxicity by the Reduction of iNOS Expression and Lipid Peroxidation

**DOI:** 10.1371/journal.pone.0029102

**Published:** 2011-12-29

**Authors:** Tai-Chun Huang, Kwok-Tung Lu, Yu-Yuan Peter Wo, Yao-Ju Wu, Yi-Ling Yang

**Affiliations:** 1 Department of Biochemical Science and Biotechnology, National Chia-Yi University, Chia-Yi, Taiwan; 2 Department of Life Science, National Taiwan Normal University, Taipei, Taiwan; University of Rome La Sapienza, Italy

## Abstract

Alzheimer disease (AD) is an age-dependent neurodegenerative disease characterized by the formation of β–amyloid (Aβ)-containing senile plaque. The disease could be induced by the administration of Aβ peptide, which was also known to upregulate inducible nitric oxide synthase (iNOS) and stimulate neuronal apoptosis. The present study is aimed to elucidate the cellular effect of resveratrol, a natural phytoestrogen with neuroprotective activities, on Aβ-induced hippocampal neuron loss and memory impairment. On adult Sprague-Dawley rats, we found the injection of Aβ could result in a significant impairment in spatial memory, a marked increase in the cellular level of iNOS and lipid peroxidation, and an apparent decrease in the expression of heme oxygenase-1 (HO-1). By combining the treatment with Aβ, resveratrol was able to confer a significant improvement in spatial memory, and protect animals from Aβ-induced neurotoxicity. These neurological protection effects of resveratrol were associated with a reduction in the cellular levels of iNOS and lipid peroxidation and an increase in the production of HO-1. Moreover, the similar neurological and cellular response were also observed when Aβ treatment was combined with the administration of a NOS inhibitor, N(G)-nitro-L-arginine methyl ester hydrochloride (L-NAME). These findings strongly implicate that iNOS is involved in the Aβ-induced lipid peroxidation and HO-1 downregulation, and resveratrol protects animals from Aβ-induced neurotoxicity by suppressing iNOS production.

## Introduction

Alzheimer's disease (AD) is a neurodegenerative disorder, which could lead to a severe dementia and affect multiple cognitive and behavioral functions. Mainly due to the worldwide aging problem, AD has accounted for about 60% of all dementia [1] and has obviously become a serious health problem to be coped with. In 2000, 25 million people in the world were diagnosed to suffer from AD, and this number is expected to increase to 114 million by 2050 [2]. Amyloid β protein (Aβ), a 39- to 43-amino acid peptide fragment derived from an amyloid precursor protein via a sequential cleavage by β- and γ- secretases, is currently believed to be tightly related to the pathogenesis of AD. Consequently, targeting on Aβ production and amyloid fibril aggregation has represented an attractive therapeutic strategy for the treatment of AD [3]. Although the precise mechanism of Aβ-induced neuronal death and AD is not well understood, Aβ neurotoxicity has been connected with various cellular impacts, including Ca^2+^ influx, glutamate accumulation, and oxidative stress [4]. Since the aging process is typically associated with an augment in the reactive oxygen species (ROS) production together with a parallel decline in the ability to defend ROS attack, oxidative stress is thus speculated to be the primary causative event that contributes to the progress of AD [5].

Nitric oxide (NO) is synthesized from L-arginine by nitric oxide synthase (NOS). It is also assumed to play a detrimental role in the pathological process of AD. In fact, three isoforms of NOS have been isolated and named by the tissues from which they were first cloned. Neuronal NOS (nNOS) and endothelial NOS (eNOS) are constitutively expressed and calcium dependent, while inducible NOS (iNOS) is expressed in response to immunologic challenge or tissue injury, but is calcium independent. In early studies, nitric oxide was reported to be excessively produced in AD [6]. It not only damaged DNA but also induced the production of peroxynitrite, which in turn destructed mitochondria and reduced ATP formation [7]. As a high level of Aβ was as well reported to activate microglia and upregulate the expression of iNOS [8], conceivably, the resulted high level of NO should have contributed to the development of neural damage in AD patients.

Resveratrol (3,5,4′-trihydroxystilbene) is a naturally occurring polyphenol, and belongs to the phytoalexin family. It can be isolated from the seeds of a variety of plant species including grapes and peanuts, and constitutes one of the valuable ingredients of red wine [9], [10]. This natural product has been reported to exhibit certain beneficial effects in the treatment of ischemia [11] and neurodegenerative disease [12]. Our recent studies also demonstrated that resveratrol could protect rats from ischemia- and 1-methyl-4-phenyl-1,2,3,6-tetrahydropyridine (MPTP)-induced neurotoxicity and free radical overloading in substantia nigra [13]. More interestingly, the cellular level of NO seems to be modulated by resveratrol, and this regulatory effect appears to be tissue (or enzyme) dependent. Resveratrol could stimulate eNOS gene expression and eNOS protein phosphorylation, leading to an increase in NO level that, by an unknown mechanism, gives rise to a protective and vasorelaxation effect [14], [15]. However, resveratrol was also demonsrated to suppress the iNOS activity by inhibiting NF-κB binding activity and post-transcriptional modification, thereby protected neurons from Aβ-induced neurotoxicity [16].

Heme oxygenase-1 (HO-1) is a 32 kDa stress induced protein that catalyzes the degradation of heme to biliverdin, Fe^2+^, and carbon monoxide [17], [18]. It also protects cells by catalyzing the catabolism of pro-oxidant metalloporphyrins to bile pigments with its free radical scavenging capabilities [19]. The activity of HO-1 is critical to oxidative challenge. In several stress-related pathological conditions, including hypoxia, atherosclerosis, and inflammation, the expression of HO-1 gene and HO-1 enzyme activity were found to be greatly enhanced [20]–[22]. Judging from the results obtained from some previous *in vitro* and *in vivo* studies, HO-1 was also suggested to be a key factor for neuroprotection. The overexpression of HO-1 resulted in a relatively higher resistance to glutamate- and H_2_O_2_-mediated oxidative damage and MPTP- or β-amyloid_1–40_-induced neurotoxicity [23]–[26]. The connection between the function of resveratrol and the level of HO-1, however, is rather paradoxical. Resveratrol was evidenced, *in vitro*, to augment cellular antioxidant defense capacity by activating HO-1 expression through the Nrf2-ARE signaling process, thereby protecting PC12 cells from oxidative stress [27]. Conversely, the effectiveness of resveratrol in ameliorating the toxicity of tBH was proved to arise from the suppression of HO-1 production [28]. The role of resveratrol on HO-1 expression apparently requires more extensive studies to clarify.

Growing bodies of evidence have supported the fact that the chronic administration of resveratrol could effectively protect various tissues against ischemic injury by reducing the free radical production [11], [13]. And, these protection activities seemed to attribute, at least partly, to the enzymatic activities and the cellular levels of iNOS and HO-1. To gain a further insight into the biological roles of resveratrol, we attempt, in this study, to elucidate the correlation between its neuroprotection effect and the iNOS and HO-1 production with a model of Aβ-induced cognitive dysfunction and neuronal damage.

## Materials and Methods

### Animals

Adult male Sprague-Dawley (SD) rats weighing 500–550 g were purchased from the BioLasco Taiwan Co., Ltd and used in the present study. Animals were housed individually, in hanging wire cages, in a temperature-controlled animal colony at 24°C with a normal 12-h∶12-h light/dark cycle. The animals had free access to food and water and were allowed to acclimate to the light/dark cycle for at least 1 week before being used in the experiments. The protocols for the animal care and treatment were approved by the Animal Care and Use Committee of National Chia-Yi University (Approval No.: 2006006).

### Experimental procedures

Based on recent *in vivo* and *in vitro* studies, the peptide Aβ_(25–35)_ was reported to perform similarly as the full-length Aβ_(1–42)_ [29] in generating the critical neurotoxic effects and amyloid fibril aggregation as in AD [30]–[33]. Investigations with the use of Aβ_(25–35)_ in animal models would provide a convenient model in understanding more AD-related mechanisms. Therefore, in this study, the peptide was used. Aβ_(25–35)_ (100 µM/5 µl)(Tocris, Bristol, UK) was first dissolved in saline and allowed to aggregate at 37°C for seven days before use. Each rat was anesthetized with sodium pentobarbitol (50 mg/kg, i.p.) and placed on a stereotaxic instrument (Stoelting, USA). The animal's skull was exposed, and a 23-gauge guide cannula (model C313G, Plastic-one Products, Roanoke, VA) was implanted in the lateral ventricle (AP, −1.8; DV, −3.5, ML, ±2.5 from bregma). To prevent clogging, a size 0 insect pin (Carolina Biological Supply, Burlington, NC) was also inserted into the cannula. After that, several screws were put onto the skull, and the assembly was cemented in place by using dental cement (Plastic-one Products, Roanoke, VA). To avoid infection, all surgically treated rats were given antibiotic, penicillin, every day for the first three days after the surgery. To each rat, Aβ_25–35_ (100 µM/5 µl)(Tocris, Bristol, UK) was injected, with 0.5 µl/min, directly into the lateral ventricle through the cannula by means of a Hamilton microsyringe and a mini-pump. The injection lasted for 10 min, and the needle, along with the syringe, was left on the injection site for another 2 min to ensure the completion of Aβ infusion. By the same procedure, Aβ was injected once a day for 7 days. To investigate the effect of resveratrol or L-NAME on the Aβ-induced neurotoxicity, resveratrol (100 µM/5 µl, i.c.v) or L-NAME (200 µg/5 µl) was injected everyday thirty minutes after the Aβ administration.

### Morris water maze

To test for the biological effects of Aβ and resveratrol on animal's spatial memory, rats were randomly separated into five groups (n = 5 for each group) and treated differently: sham group, Aβ group (Aβ, 100 µM/5 µl/day, 7 days, i.c.v), Aβ and resveratrol (100 µM/5 µl/day, 7 days, i.c.v) group, Aβ and L-NAME (200 µg/5 µl/day, 7 days, i.c.v) group, and resveratrol (100 µM/5 µl/day, 7 days, i.c.v) group. The experimental device for the test was a circular tank (180 cm diameter; 60 cm height) filled with water at temperature 23±2°C. At the midpoint of one quadrant, 1 cm below the water level, was placed a target platform (10×10 cm). It was made to have equal distance from the center to the edge of the tank. The water tank was placed in a test room with many major visual cues. The acquisition training session was performed 7 days starting from the injection of Aβ or other chemicals. Rats were arbitrarily left in the tank, facing the wall, and allowed to swim freely. They were gently directed to the escape platform if they could not find the platform within 2 minutes. The animals were then allowed to stay on the platform for 2 minutes. The procedure was repeated 4 times a day with each animal, and the escape latency time was recorded [34].

### Western blot

After the behavioral test, the rats from each group were decapitated and their brains were rapidly removed. Following dissection, each hippocampus was weighed and rapidly homogenized in a 6 volumes of ice-cold homogenizing buffer (T-PER, Pierce, USA). Total hippocampal proteins were isolated by centrifugation and electrophoretically separated on an 8% sodium dodecylsulfate (SDS) polyacrylamide gel. The resolved proteins on the gel were electro-blotted onto a polyvinylidene difluoride membrane. Immunoblotting analysis was then performed by using monoclonal antibody anti-iNOS (1∶2000 dilution) or anti-HO-1 (1∶5000 dilution) (Cell Signaling, USA), as the primary antibody. The ultimate signals from each analysis were visualized with an enhanced chemiluminescence assay (RPN 2108; Amersham International, Amersham, UK). The intensities of the detected signals were analyzed by semiquantitative densitometry in conjunction with AlphaEase software (Alpha Innotech Corp., CA) [35].

### Determination of neuromuscular coordination

Rota-rod accelerating test was also performed to each animal. It was to examine the possible defects in neuromuscular coordination that might occur on the chemically treated rats [13]. Before the stereotaxic surgery, each rat was placed in a rota-rod apparatus (Ugo Basile, Varese, Italy) and subjected to accelerating test. The rat was placed on the rotating rod (at the slowest speed, 4 rpm) for 2 minutes. The rats that could not hold on the rod for more than 2 minutes were excluded from the further experiments, including stereotaxic surgery and chemical treatment. For the qualified rats that were used in chemical treatment, starting from the 6^th^ day after surgery, the rats were trained per day as described above for 2 days. At day 7 after surgery, the rotational speed of the rod was then switched to its maximum speed of 40 rpm, and the length of the time rats could grasp at the rod was measured. The test score is the average number of seconds that rats could hold onto the rod per trial. The variation in rota-rod performance among rat groups was used to evaluate the impairment of the motor coordination [36].

### Malondialdehyde (MDA) assay

In this study, we also perform a malondialdehyde (MDA) assay to evaluate the level of lipid peroxidation in hippocampus derived from the rats that were treated with different chemicals. The rats were sacrificed after surgery and behavioral test, and the hippocampus were carefully dissected and immediately stored at −70°C. The content of MDA was determined by a modified procedure of Li et al [37]. Each hippocampus section (100 mg) was homogenized in 1 ml of 10 mM phosphate buffer (pH 7.4). After centrifugation at 12,000×g for 20 min, the MDA content in the supernatant was assayed by the thiobarbituric acid (TBA) reaction, and the resulted colored product was measured at 535 nm with spectrometer. The levels of TBA reactive species were indicated as nmol/mg protein.

### Histology evaluation of neuronal damage

Another set of chemically treated rat groups (n = 4, each group) were sacrificed, at day 7 of the surgery with an overdose of pentobarbitol (100 mg/kg sodium pentobarbital, i.p.), and subsequently perfused transcardically with 0.9% NaCl and 10% formalin. Following formalin perfusion, the rats were decapitated and their brains were removed from the skulls and embedded in paraffin blocks. Coronal sections (2 µm) through the brain were stained with hematoxylin and eosin (H&E) for microscopic evaluation. Each hemisphere was studied independently without the examiner knowing the experimental conditions.

### Statistical analysis

All data obtained in this experiment were expressed as mean ± SEM. Significance of changes of Aβ, iNOS and HO-1 was determined by the nonparametric Mann-Whitney U test. Escape latency and retention time were also compared among groups by the nonparametric Mann-Whitney U test. We considered *p*<0.05 to be statistically significant.

## Results

### Aβ administration significantly increased the Aβ accumulation in hippocampus and resveratrol treatment decreased the hippocampal Aβ accumulation

**Figure 1:**
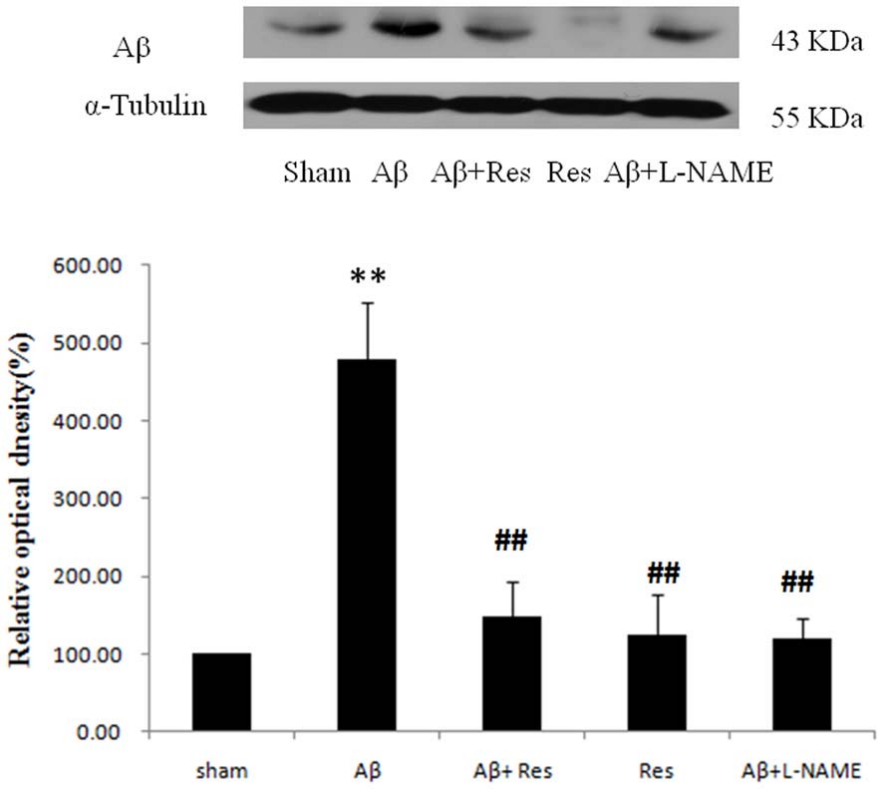
Effects of resveratrol on hippocampal Aβ accumulation. (A) Western blot analysis on the Aβ protein from sham rats (sham), the rats with Aβ_25–35_ administration for seven days (Aβ), the rats given with Aβ_25–35_ and resveratrol (Aβ+Res), the rats administered with resveratrol only (Res), and the rats combined with Aβ_25–35_ and L-NAME administration (Aβ+L-NAME); (B) Relative Aβ protein quantified as compared with sham group (normalized to 100%). Data were indicated as mean ± SEM values (n = 5). **p<0.01 was considered significantly different from sham values by Mann-Whiney U test. ##*p*<0.01 was considered significantly different from Aβ values by Mann-Whiney U test.

To establish a plausible rat model of AD, we first examined, by immunobloting, whether the Aβ_25–35_ peptide administration would induce the Aβ accumulation in hippocampus [33]. According to the manufacturer, the band near 43 kDa represents the expression of Aβ in rats. As compared with that of sham group (Figure 1), a more than 4.5-fold increase in Aβ protein level was observed in the rat group with Aβ peptide administration. Such over-aggregation of hippocampal Aβ did not occur if the rats were injected with resveratrol alone. When combined with Aβ peptide treatment, resveratrol actually could significantly attenuate the Aβ accumulation that presumably would be induced by Aβ peptide injection. Similar to resveratrol, the drug, L-NAME, a NOS specific inhibitor, also appeared to be a potent agent in preventing Aβ accumulation.

### Resveratrol improved Aβ-induced spatial memory deficiency

**Figure 2:**
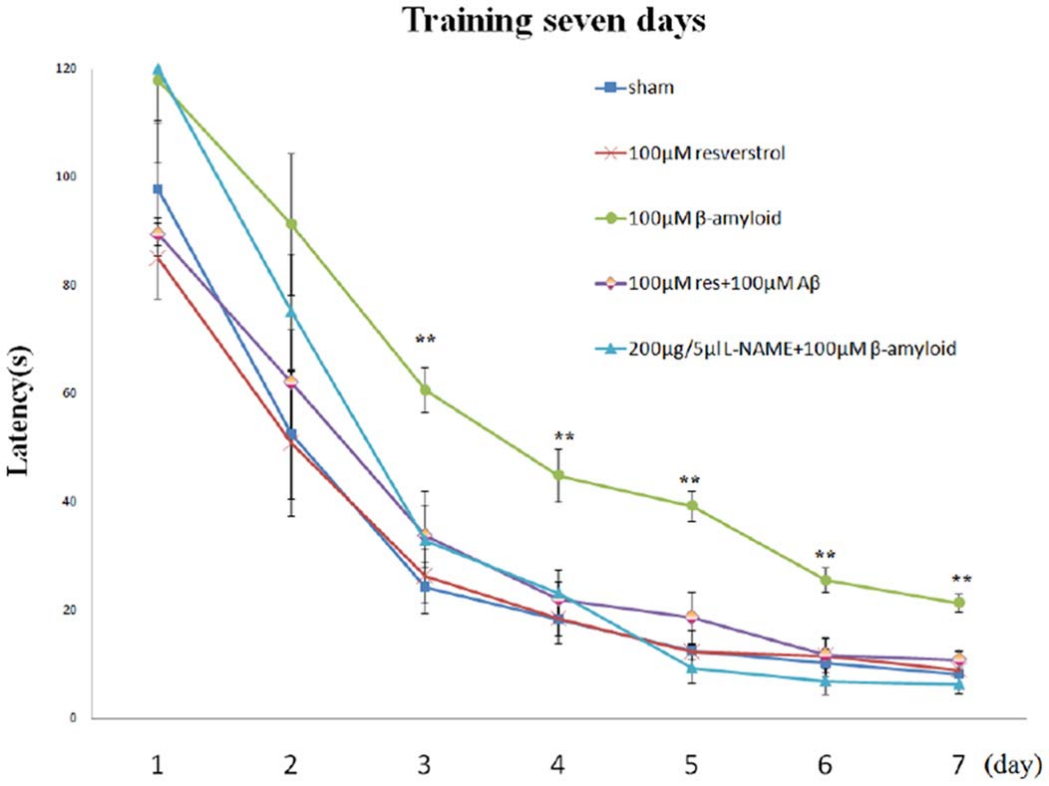
Effects of resveratrol or L-NAME on escape latency in the training trials of the water maze task. The different treated rats were subjected to the analysis: the rats were infused icv with vehicle (sham), the rats with Aβ_25–35_ administration for seven days (Aβ), the rats with the injection of Aβ_25–35_ and resveratrol (Aβ+Res), the rats administered with resveratrol only (Res), and the rats with Aβ_25–35_ and L-NAME administration (Aβ+L-NAME). Data were represented as mean ± SEM values (n = 5). **p<0.01 was considered significantly different from sham values by Mann-Whiney U test.

**Figure 3:**
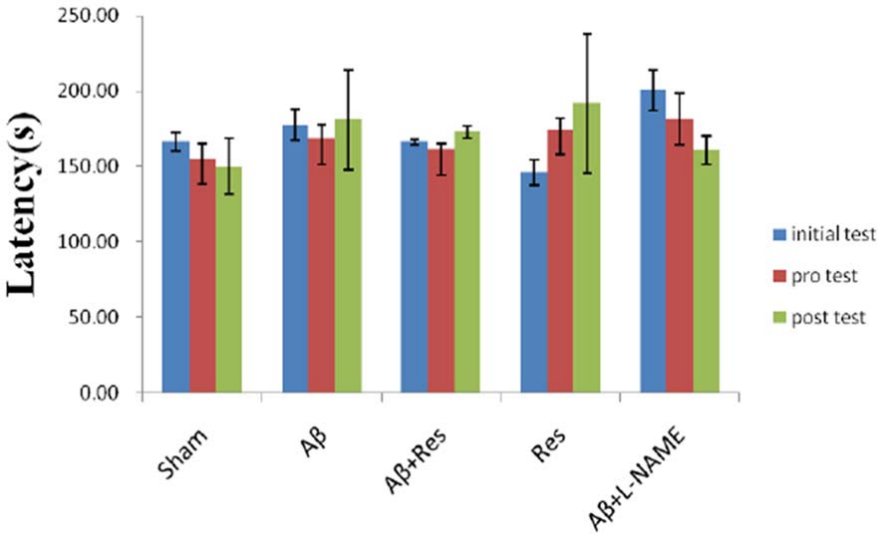
Effects of Aβ, resveratrol and L-NAME administration on the retention time of rota-rod. The training procedure is as described in Materials and Method. Bars represent mean ± SEM values (n = 5).

Besides the suppression of Aβ accumulation, the role of resveratrol was also extended to exhibit a beneficial effect against the Aβ-induced spatial memory deficiency. In this experiment, the escape latency was recorded after the rats were injected with Aβ peptide. In training trials, the escape latency time on the last day of training (the 7th day of surgery) was 8.21±2.13 seconds for sham group, 21.21±1.91 seconds for Aβ (100 µM) treated group, and 10.71±1.75 seconds for the rat group with a combined injection of Aβ and resveratrol (Figure 2). The escape latency time was significantly prolonged in Aβ treated rat group as compared with that of sham group (p<0.01). However, there was no significant difference between sham and Aβ + resveratrol groups (p = 0.979). As compared with Aβ group, Aβ+ resveratrol group rats spent a much shorter time on the target quadrant to reach the escape platform, suggesting that resveratrol administration was rather effective in improving the spatial recognition and memory, which were obviously disrupted by Aβ. These results, however, could not exclude the possibility that the given surgery might also affect rat swimming ability and influence the measurement of escape latency time. To rule out the possibility, the swimming speed of rats was also measured. The average swimming velocity of sham, Aβ treated, and Aβ combined with resveratrol groups of rats were 23.38±0.77, 22.3±1.65 and 21.26±1.53 cm/sec, respectively. In fact, no obvious defect in the swimming ability, from the tests of the Morris Water Maze and the total square crossing, was observed among different rat groups. In addition, based on the results of rota-rod tests on the rats' neuromuscular function, there was again no apparent alteration among the differently treated rat groups (Figure 3). The latency times of sham, Aβ, Aβ combined with resveratrol, and Aβ combined with L-NAME were 150.21±18.54, 180.25±32.97, 172.79±4.17, and 160.67±9.43, respectively. All these evidences pointed to the fact that, except for the behavior alteration observed in spatial memory test, there was little motor deficit occurred in the animals with or without various chemical treatment.

### Resveratrol reversed Aβ-induced iNOS expression

**Figure 4:**
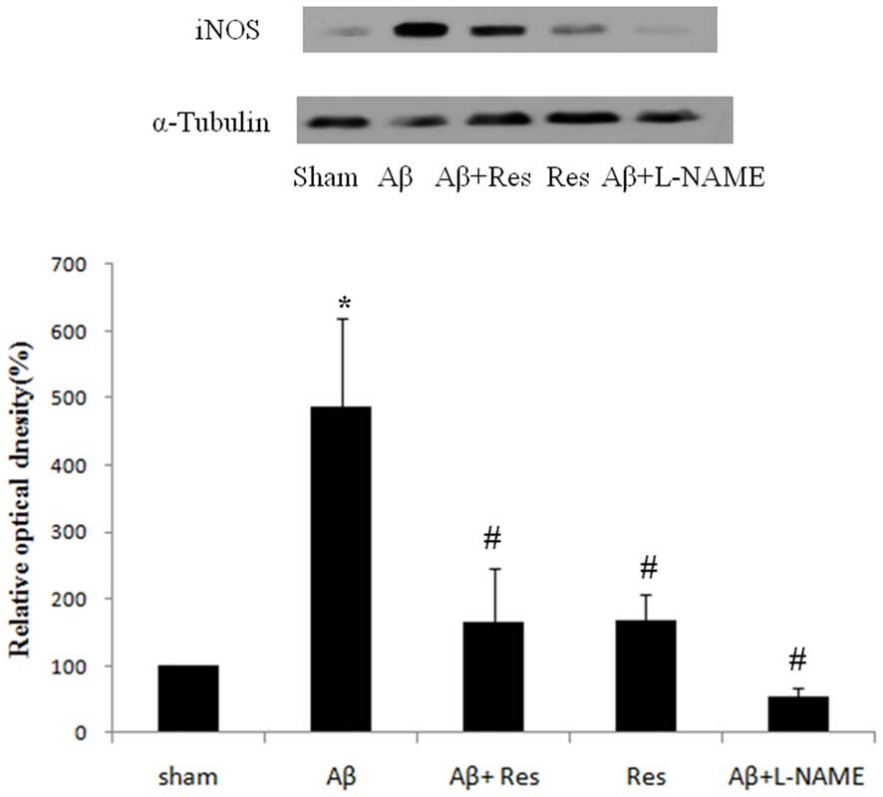
Drug effects on the hippocampal iNOS expression. (A) Western blot analysis on sham rats (sham), the rats with Aβ_25–35_ administration for seven days (Aβ), the rats with injection of Aβ_25–35_ and resveratrol (Aβ+Res), the rats administered with resveratrol only (Res), and the rats injected with Aβ_25–35_ and L-NAME (Aβ+L-NAME); (B) Relative iNOS level quantified as compared with sham group (normalized to 100%). Data were represented as mean ± SEM values (n = 5). *p<0.05 was considered significantly different from sham values by Mann-Whiney U test. #*p*<0.05 was considered significantly different from Aβ values by Mann-Whiney U test.

To unravel the connection between iNOS and the Aβ-induced neurotoxicity, animals were treated with Aβ, Aβ plus resveratrol, or Aβ plus L-NAME, and the iNOS level in each rat hippocampus was analyzed by Western blotting. As shown in Figure 4, the production of iNOS was markedly stimulated (∼5 folds) by Aβ administration. Resveratrol treatment reversed the Aβ-induced iNOS overexpression, and L-NAME conferred a similar but more profound effect, as that of resveratrol, on iNOS production suppression. These results clearly demonstrated that resveratrol could effectively suppress iNOS expression that was stimulated by Aβ.

### Resveratrol enhanced HO-1 expression in hippocampus

**Figure 5:**
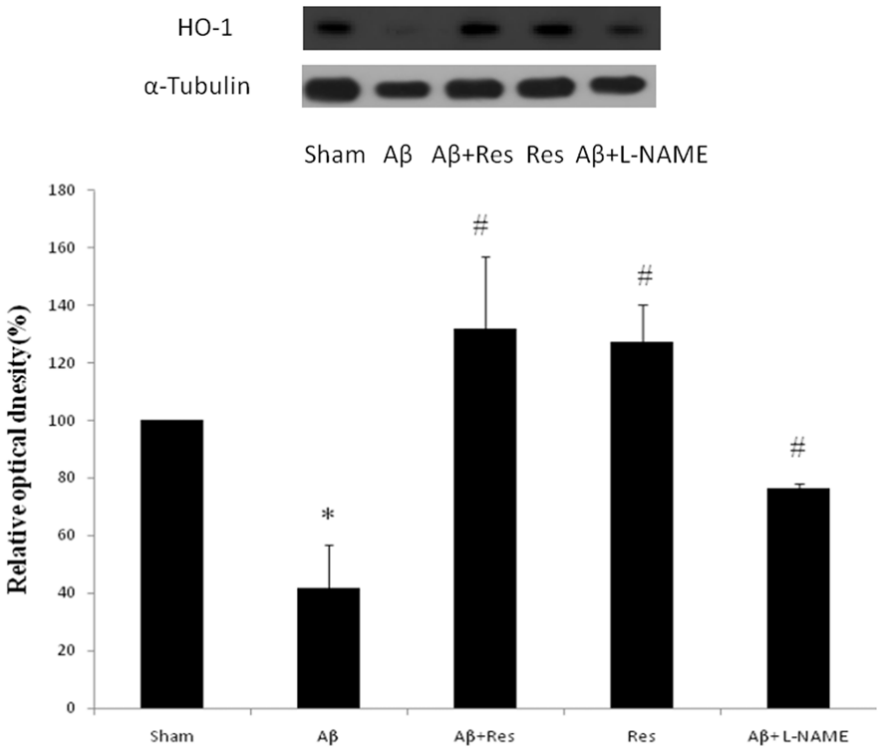
Drug effects on the hippocampal HO-1 expression. (A) Western blot analysis on sham rats (sham), the rats with Aβ_25–35_ administration for seven days (Aβ), the rats with injection of Aβ_25–35_ and resveratrol (Aβ+Res), the rats administered with resveratrol only (Res), and the rats injected with Aβ_25–35_ and L-NAME (Aβ+L-NAME); (B) Relative iNOS level quantified as compared with sham group (normalized to 100%). Data were represented as mean ± SEM values (n = 5). *p<0.05 was considered significantly different from sham values by Mann-Whiney U test. #*p*<0.05 was considered significantly different from Aβ values by Mann-Whiney U test.

Since oxidative stress has been implicated as a major causality of neuronal damage in AD, and HO-1 appears to be a critical enzyme to defend neurons against oxidative stress, the detection of hippocampal HO-1 level was also included in the list of our investigation. In comparison with the sham group, the rats injected with Aβ peptide revealed an apparent decline in the hippocampal HO-1 expression (∼40% of sham) (Figure 5). Resveratrol, however, caused a ∼30% increase in HO-1 production with or without the co-treatment with Aβ peptide. Interestingly, the NOS inhibitor L-NAME, which has never been reported to modulate HO-1 gene expression, also depressed the Aβ-induced inhibition in HO-1 production, though it was not as effective as resveratrol.

### Resveratrol suppressed lipid peroxidation

**Figure 6:**
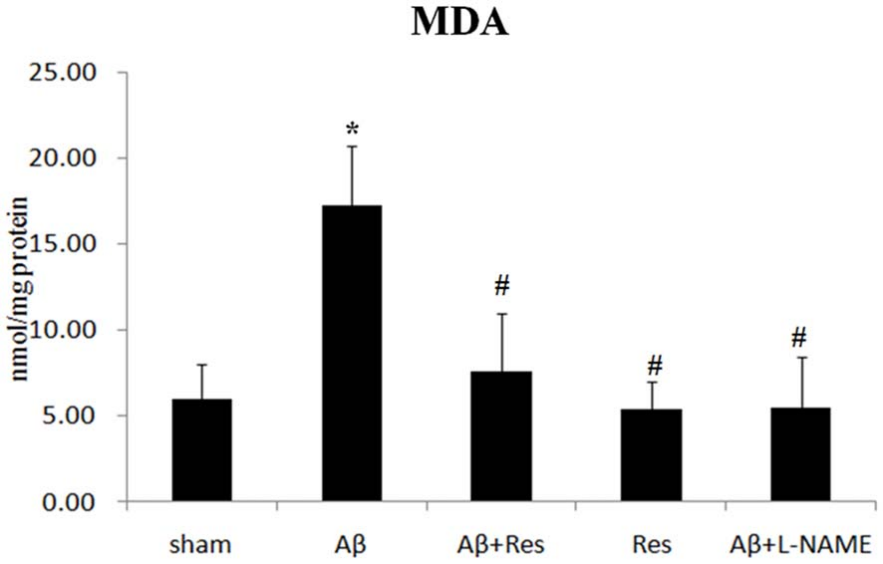
Drug effects of resveratrol on the hippocampal MDA level. The rats were infused icv with vehicle (sham), the rats with Aβ_25–35_ administration for seven days (Aβ), the rats injected with Aβ_25–35_ and resveratrol (Aβ+Res), the rats administered resveratrol only (Res), and the rats injected with Aβ_25–35_ and L-NAME (Aβ+L-NAME). Data were represented as mean ± SEM values (n = 5). *p<0.05 was considered significantly different from sham values by Mann-Whiney U test. #*p*<0.05 was considered significantly different from Aβ values by Mann-Whiney U test.

 To further bear out the anti-oxidative activity of resveratrol, the level of lipid peroxidation was assayed by measuring the level of hippocampal MDA. The detected average MDA amount from sham rats was 6.0±2.0 ng/mg protein. It was remarkably lower than that from the rats treated with Aβ peptide (17.3±3.5 ng/mg protein(p = 0.005)). When the resveratrol was given alone or together with the Aβ peptide, the average amount of MDA declined to 5.4±1.6 or 7.6±3.4 ng/mg protein respectively. Similar to the effect of resveratrol, the high MDA level resulted from the stimulation of Aβ could be effectively reduced by L-NAME to 5.5±3.0 ng/mg protein (Figure 6).

### Resveratrol protected rats from Aβ-induced neuronal death

**Figure 7:**
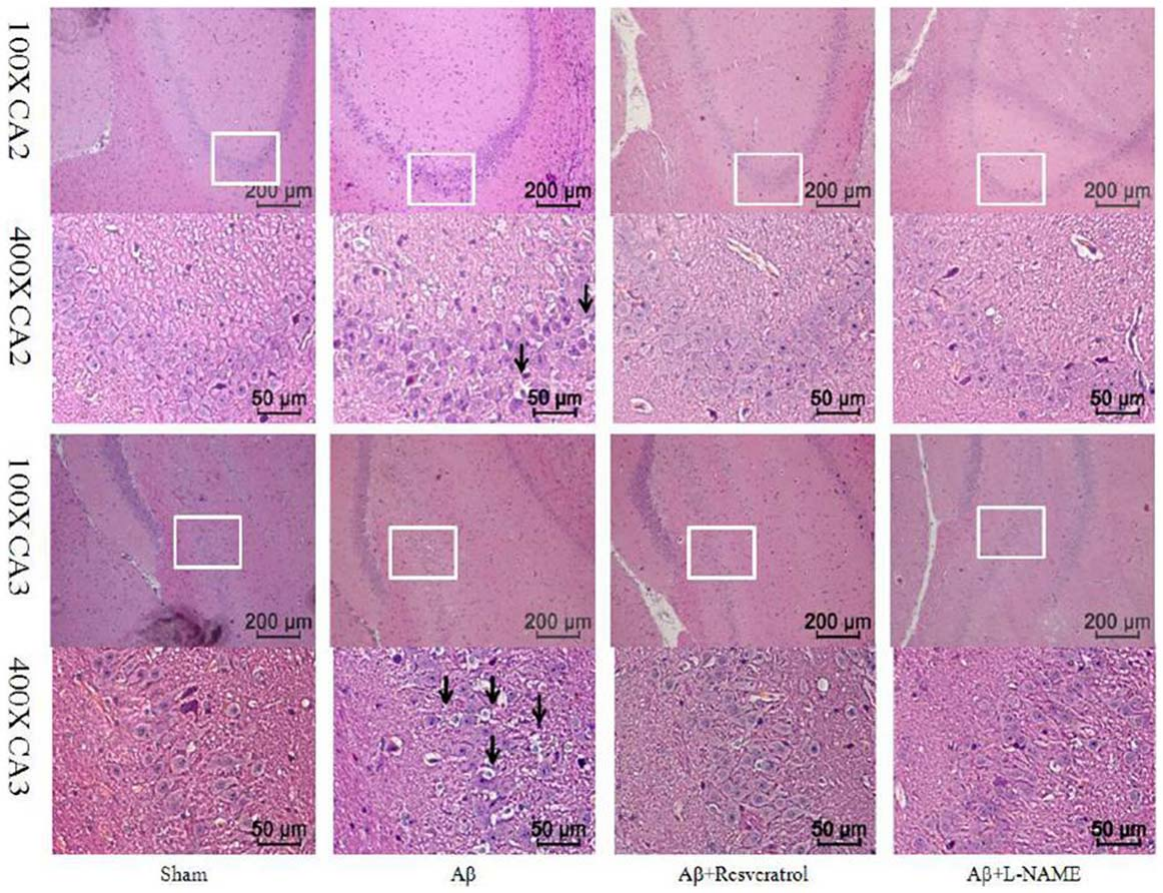
Photomicrographs showing the morphology of the hippocampal CA1 (the first and second panel) and CA3 (the third and fourth panel). The rats infused icv with vehicle (sham), the rats with Aβ_25–35_ administration for seven days (Aβ), the rats injected with Aβ_25–35_ and resveratrol (Aβ+Res), the rats injected with Aβ_25–35_ and L-NAME (Aβ+L-NAME) (n = 4). The Aβ-induced neuronal shrinkage and damage are indicated as arrows. The bar scale of the first and third panel are 200 µm and the bar scale of second and fourth are 50 µm.

Resveratrol also reduced the severe Aβ-induced neuronal damage. The histological photographs for the examination of neuronal damage from the differently treated rat groups are shown in Figure 7. The rats subjected to Aβ injection revealed a severe neuronal damage at hippocampus with much more shrunk and damaged neurons than that of sham group. The extent of Aβ-induced neuronal damage was significantly decreased at the hippocampus derived from the rats treated with Aβ plus resveratrol. Similarly, a much lower level of hippocampal neuron damage was observed in the rats treated with Aβ plus L-NAME.

## Discussion

In the present study, we have extensively investigated the neuroprotective activity of resveratrol by using a rat model of Alzheimer's disease. A chronic (7 days) i.c.v. injection of 100 µM Aβ peptide could result in an impaired learning and memory behavior on rats. This memory impairment apparently was associated with lipid peroxidation elevation, HO-1 downregulation, iNOS overexpression, and severe neuronal loss. By using the NOS inhibitor, L-NAME, we have clarified the role of iNOS in the Aβ-induced memory deficiency, lipid peroxidation generation, and neuronal damage. The major findings from this study also have shed considerably more light on the biological role of resveratrol. It could provide beneficial effects on the recovery of learning and memory, suppress the production of hippocampal iNOS, prevent the accumulation of lipid peroxidation products, and enhance the expression of HO-1. As the chronic administration of resveratrol also protected animals from Aβ-induced neuronal loss, it is reasonably speculated that the improvement in spatial memory by resveratrol might be ascribed to its effectiveness in reducing the levels of oxidative stress and the Aβ-induced neuronal loss.

Based on some rodent behavioral studies, including the Morris water maze and passive avoidance [38], administration of Aβ_25–35_ was found to provoke a significant memory impairment. In order to mimic the progress of AD, we infused Aβ peptide into lateral ventricle daily for seven days. This rodent AD model seems to be plausible as Aβ peptide induction indeed caused the Aβ accumulation and spatial memory impairment. Our results are well consistent with that of other studies [39]–[40]. With this model, we have one step further demonstrated that the chronic treatment of resveratrol (100 µM, i.c.v.) could effectively eliminate the formation of Aβ in hippocampus and protect the rat from the impairment of Aβ-induced spatial memory. The observed effect of resveratrol on the avoidance of neurotoxic Aβ accumulation conceivably has contributed greatly to the improvement of rat's memory.

Nitric oxide, a short-lived messenger, has been evidenced to be involved in the physiological and pathophysiological development of Alzheimer's disease [41], [42]. Our results have indicated that the synthesis of iNOS was greatly stimulated by Aβ injection. This presumable would lead to a drastic increase in the concentration of NO in hippocampus. Furthermore, such Aβ-induced stimulation in iNOS production could be reversed by resveratrol as well as the NOS inhibitor, L-NAME. In fact, the similar effect of resveratrol on Aβ-induced iNOS expression was also observed in C6 glioma cells [43]. According to previous studies, the modulation of resveratrol on both Aβ accumulation and iNOS regulation may not be so surprising. The activation of iNOS gene by Aβ is controlled by the transcription factor NF-κB [43], which is known to regulate iNOS transcription by binding to the regulatory region of iNOS gene [44]. In addition, Aβ treatment was reported to result in the activation of NF-κB in various brain regions and cell types [45], [46], and resveratrol was shown to markedly attenuate the Aβ-induced NF-κB nuclear translocation [43], [47]. Based on these, it is suggested that the regulation pathway triggered by resveratrol is to decrease the Aβ accumulation, which in turn suppresses Aβ-induced NF-κB translocation, or activation, and leads to the downregulation of iNOS. More interestingly, the decrease in iNOS by resveratrol might confer a further decline in Aβ accumulation as blocking the iNOS expression (i.e. by L-NAME) appeared to reversely suppress the accumulation of Aβ (Figure 1).

As the neuroprotective role of HO-1 has been described in earlier studies [31], it has raised our interest in investigating the possible cellular modulation of resveratrol on the gene expression of *HO-1*. HO-1 overproduction was detected in hippocampus and cortex in AD patients, but not in normal control preparations [48]. In addition, *HO-1* gene expression was found to be co-localized with neurons, astrocytes, and gliocytes [49], and its gene upregulation was believed to be a relatively early event in the pathogenesis of AD [49], [50]. A piece of direct evidence for this was proposed based on a transgenic mice model, in which the long-term overexpression of HO-1 was found to enhance iron loading and the phosphorylation (Ser199/202/396) and aggregation of tau [51]. In contradistinction to the enhanced HO-1 expression documented from AD patients, the suppression of HO-1 production in the cerebral spinal fluid and choroids plexus epithelials cell were also reported [26], [49], [50]. In contrast to that from the previous *in vitro* experiment with PC12 cells [52], our results showed that the Aβ chronic treatment *in vivo* induced a decrease, rather than an increase, in the HO-1 expression. And, this could be reversed by the co-treatment with resveratrol. As a matter of fact, the results obtained by using resveratrol in the current study were similar to that of another study by using atorvastatin [53]. In that, a long-term treatment with atorvastatin (80 mg/d for 14.5 months) appeared to upregulate HO-1, and the increase in HO-1 production was associated with a lower discrimination learning error scores in aged beagles [53]. It is of particular interest that, in analogy to the effect conferred by resveratrol, L-NAME also showed a depression activity on *HO-1* expression, suggesting that *HO-1* gene regulation is, at least in part, mediated by iNOS.

Some strong evidences, including that obtained from several clinical and basic researches, have highlighted to the fact that free radicals and oxidative stress are associated with aging and AD. Free radical attack might cause lipid peroxidation, protein peroxidation, DNA peroxidation, and other oxidative stress, and conceivably is the central causality in age-related diseases [54]. It is thus believed that some antioxidants should bear the potential to attenuate the cognitive decline in AD and retard the progress of the disease [55]. In the current study, we have measured the level of the MDA derived from the lipid membrane oxidation to reflect the overall degree of free radical attack. Our results revealed a fact that resveratrol could reverse the Aβ-induced MDA overproduction. It should also be noted that L-NAME treatment also gave a similar effect as that of resveratrol in decreasing the MDA level.

In conclusion, the spatial memory impairment and neuronal death occurred in the progress of AD is strongly correlated to the expression of iNOS. The level of iNOS mediates the Aβ-induced lipid peroxidation and HO-1 expression, and resveratrol protects rats from Aβ-induced neurotoxicity through the downregulation of iNOS.
